# High-speed flat-detector computed tomography for temporal bone imaging and postoperative control of cochlear implants

**DOI:** 10.1007/s00234-022-02940-x

**Published:** 2022-04-12

**Authors:** Felix Eisenhut, Lava Taha, Michael Manhart, Vivian Thimsen, Konstantinos Mantsopoulos, Heinrich Iro, Joachim Hornung, Arnd Dörfler, Stefan Lang

**Affiliations:** 1 Department of Neuroradiology, University Hospital Erlangen, Friedrich-Alexander University Erlangen-Nuremberg, Schwabachanlage 6, 91054 Erlangen, Germany; 2 Department of Otorhinolaryngology, Head and Neck Surgery, University Hospital Erlangen, Waldstraße 1, 91054 Erlangen, Germany; 3 Advanced Therapies, Innovation, Siemens Healthcare GmbH, Siemensstraße 1, 91301 Forchheim, Germany

**Keywords:** Flat-detector computed tomography, Temporal bone, Anatomy, Cochlear implant, Postoperative control

## Abstract

**Purpose:**

Flat-detector computed tomography (FD-CT) is the standard for cochlear implant (CI) imaging. FD-CT systems differ in technical characteristics. Our aim was an evaluation of two different FD-CT generations with different protocols and hardware regarding image quality, radiation dose, and scan time.

**Methods:**

Two temporal bone specimens (− / + CI = TB_0_/TB_1_) were scanned using three different scanners: two FD-CT systems with different scanning protocols (standard FD-CT: 20 s 70 kV, 20 s 109 kV; high-speed FD-CT [HS-FD-CT]: 7 s 109 kV, 9 s 109 kV, 14 s 72 kV) and MS-CT (5 s 120 kV). Acquired datasets were evaluated in consensus reading regarding qualitative and quantitative parameters: addressing CI- and cochlea-specific parameters, cochlea delineation, lamina spiralis ossea visibility, distinction of single CI electrodes, determination of intracochlear implant position, stapes delineation, and mastoidal septation were assessed. Addressing protocol-specific parameters, radiation dose (dose-length-product/DLP), and scan time were assessed.

**Results:**

Two HS-FD-CT protocols (14 s/9 s) provide higher or equivalent diagnostic information regarding CI- and cochlea-specific parameters compared to both standard FD-CT protocols. The fastest HS-FD-CT protocol (7 s)—providing inferior diagnostic information compared to all other FD-CT protocols—still exceeds MS-CT. The highest DLP was recorded for the 14 s HS-FD-CT protocol (TB_1_ = 956 mGycm); the lowest DLPs were recorded for the 7 s HS-FD-CT protocol (TB_0_ = 188 mGycm) and for MS-CT (TB_0_ = 138 mGycm), respectively. HS-FD-CT allows a significant reduction of scan time compared to standard FD-CT.

**Conclusion:**

High-speed FD-CT improves visualization of temporal bone anatomy and postoperative assessment of CIs by combining excellent image quality, fast scan time, and reasonable radiation exposure.

## Introduction

Cochlear implants (CI) revolutionized the treatment of patients with sensory hearing loss providing a viable option to restore hearing [1, 2]. In this context, the optimal intracochlear implant position achieving the best postoperative outcome for a patient’s speech perception and the highest rates of hearing preservation remains a widely debated issue [3–8]. To exclude a possible electrode array tip fold-over or insertion trauma (i.e. the electrode shifts from the scala tympani to the scala vestibuli) deteriorating the patient’s benefit of the implant [9, 10], postoperative radiologic CI control is part of clinical routine.

Imaging of the temporal bone with its diminutive anatomical structures remains a radiologic challenge, even more complicated by metal artifacts due to the electrode array of the CI. Combining impressive image quality and lower radiation dose than multi-slice computed tomography (MS-CT) with an affordable price, cone beam computed tomography (CBCT)—a system equipped with an X-ray source and a contralateral mounted flat panel detector rotating around the patient’s head for image acquisition [11]—is the preferred image modality not only for postoperative CI control [12, 13] but also for dental and maxillofacial imaging [14]. However, in contrast to its common availability in smaller hospitals, dedicated CBCT scanners are still not widely available in specialized departments of neuroradiology [15].

In this context, the more versatile and in most departments of neuroradiology available modern angiography systems with c-arm-mounted flat-panel detectors also allow the acquisition of volumetric CT datasets and demonstrated promising result for imaging of the temporal bone by taking advantage of excellent spatial resolution, low susceptibility for metal artifacts and a reasonable patient’s radiation exposure [16–21]. However, these standard flat-detector computed tomography (FD-CT) systems (depending on the manufacturer) usually require long scan times (e.g. up to 20 s). Especially in cases of early postoperative controls, extensive scan times might be associated with motion artifacts with consecutive substantial reduction of diagnostic value.

In this context, new high-speed FD-CT (HS-FD-CT) systems harbouring high-resolution detectors and faster, high-speed C-arms arouse interest [22] as these parameters are of relevance for further improvement of temporal bone imaging. Therefore, clinical application of HS-FD-CT might be a promising approach to reduce the susceptibility of this examination to motion artifacts by shortening the scan time and to increase diagnostic information, respectively.

Thus, using two temporal bone specimens, we assess different HS-FD-CT protocols applicable in the clinical routine regarding image quality (IQ), radiation dose, and scan time and compare them to both standard FD-CT protocols and MS-CT. Moreover, we want to propose reasonable applications of each assessed protocol in typical clinical settings and the daily routine.

## Methods

### Specimen

Two temporal bones (TB_0_/TB_1_) were dissected from the skull of a normal hearing patient who died from causes unrelated to ear or skull disease. Written informed consent was obtained to use the body for clinical research. One temporal bone (TB_1_) was implanted with a *SYNCHRONY Flex 28* cochlear implant (MED-EL Elektromedizinische Geräte Gesellschaft m.b.H, Innsbruck, Austria) at the department of Otorhinolaryngology, Head and Neck Surgery of our hospital. The study was performed according to the Declaration of Helsinki and the European Guidelines for Good Clinical Practice. Additional ethical review was not required for this analysis in accordance with local legislation (BayKrG Art. 27 (4)) and institutional requirements.

### Acquisition

**Fig. 1:**
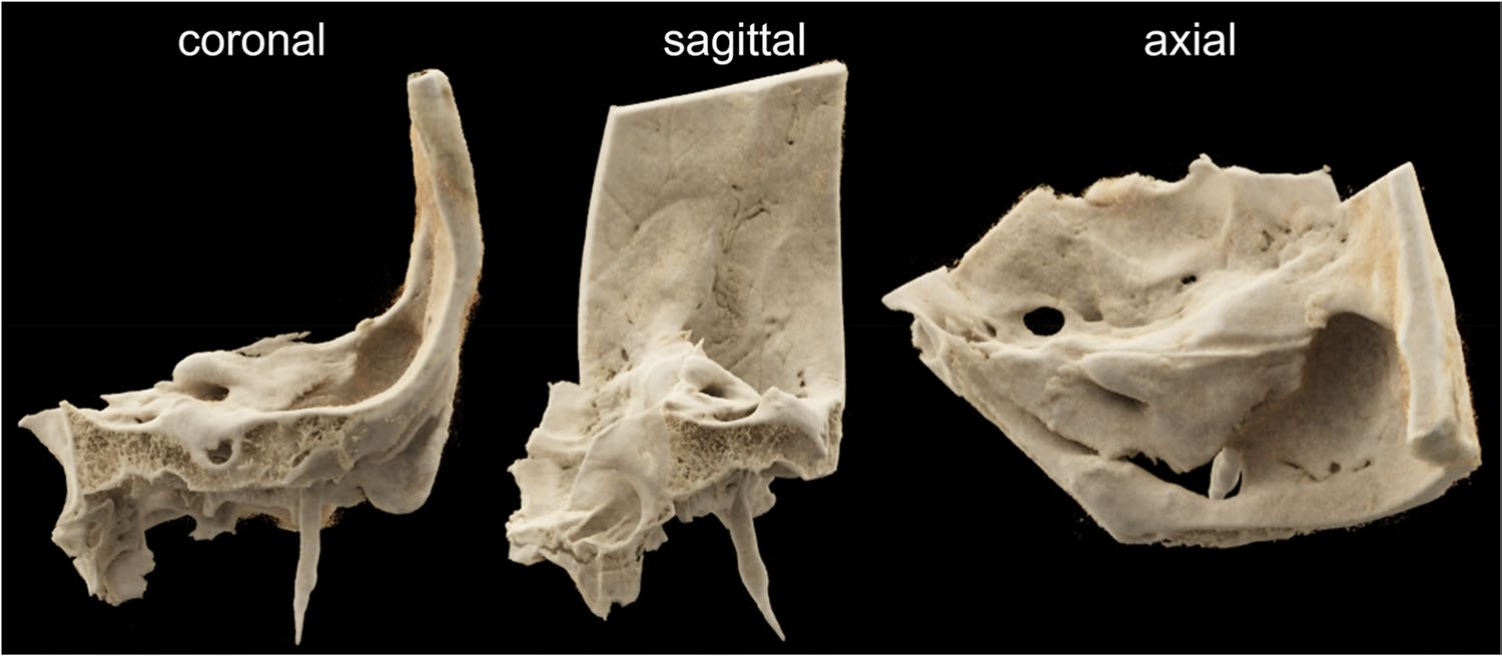
Exemplary volume rendering technique reconstruction of a scanned temporal bone specimen

Each specimen was scanned on a MS-CT and two different FD-CT systems with different scanning protocols. For scanning protocol parameters, see Table 1. Figure 1 shows a volume rendering technique reconstruction of one of our temporal bone specimens.

**Table 1:** Technical parameters of the MS-CT, FD-CT, and HS-FD-CT with different scanning protocols

	MS-CT	FD-CT 20 s 70 kV	FD-CT 20 s 109 kV	HS-FD-CT 7 s	HS-FD-CT 9 s	HS-FD-CT 14 s
Scan time (sec)	5	20	20	7	9	14
Scan length (cm)	5	5	5	5	5	5
Slice thickness (mm)	0.4	0.06	0.06	0.06	0.06	0.06
Rotation angle	360°	200°	200°	200°	200°	200°
kV	120	70	109	109	109	71.9
Pulse width (ms)	-	3.4	3.2	3.5	3.5	6.8
mAs	190	273	70	71.2	78.2	430.2
Frame rate	-	25	25	80	60	35
matrix	512 × 512	512 × 512	512 × 512	512 × 512	512 × 512	512 × 512
Binning	-	2 × 2	2 × 2	4 × 4	2 × 2	1 × 1

*MS-CT*, multislice CT; *FD-CT*, flat-detector computed tomography; *HS-FD-CT*, high-speed flat-detector computed tomography

**Fig. 2:**
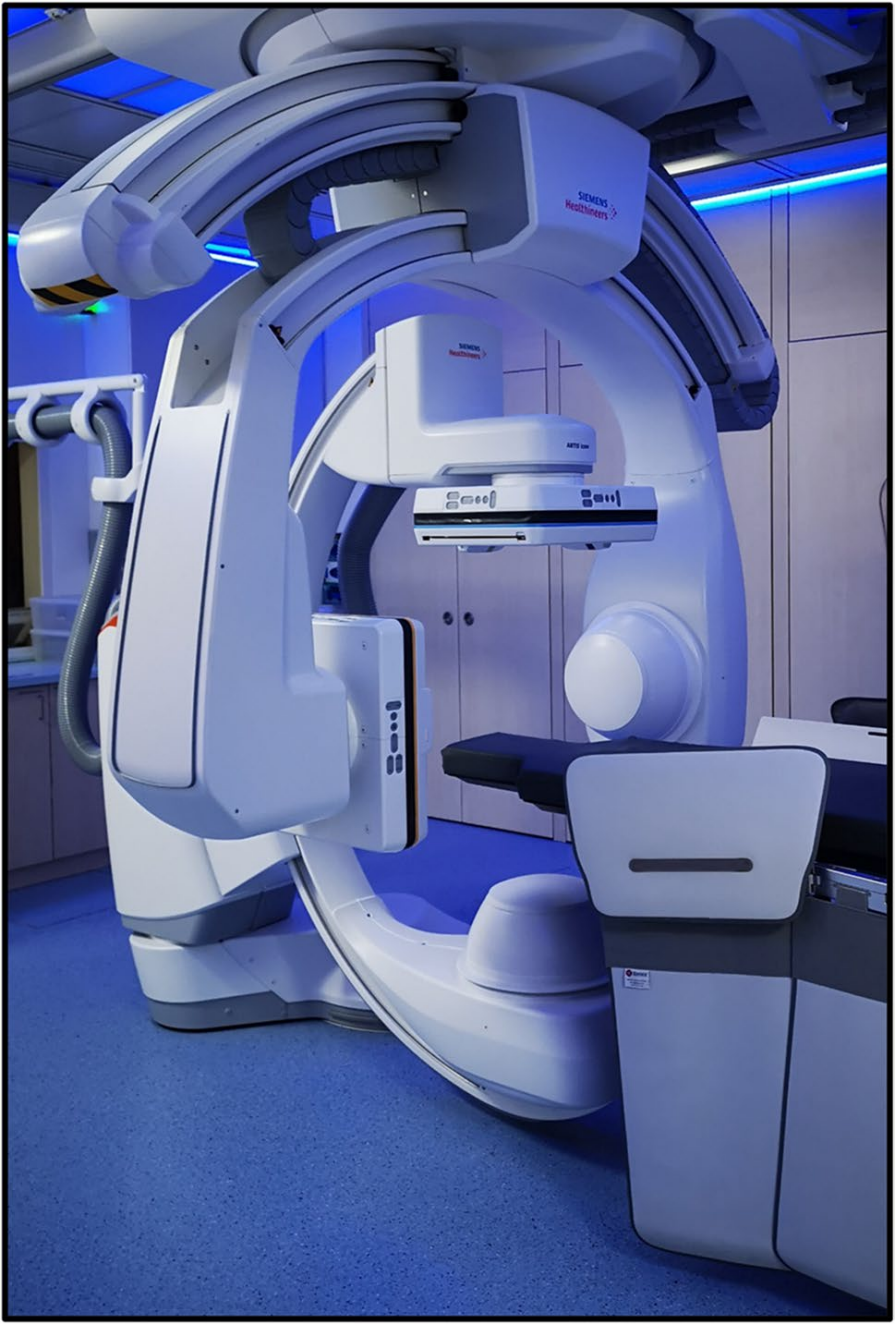
HS-FD-CT scanner: biplane angiography system with mounted FD-CT

MS-CT was performed at a 128-row scanner (Somatom Definition AS + , Siemens Healthcare GmbH, Erlangen, Germany). FD-CT was performed at a monoplane angiographic system (ARTIS Zeego, Siemens Healthcare GmbH, Erlangen, Germany, May 2008) using a 40 × 30 cm flat panel detector. The following scanning protocols were used: 20 s 70 kV, 20 s 109 kV. HS-FD-CT was performed at a biplane angiographic system (ARTIS Icono, Siemens Healthcare GmbH, Erlangen, Germany, February 2020) using a 40 × 30 cm flat panel detector. The following scanning protocols were used: 7 s, 9 s, and 14 s. Figure 2 shows the biplane angiographic system used for HS-FD-CT acquisition.

### Postprocessing

FD-CT data was transferred to a dedicated workstation (Leonardo, Siemens Healthcare GmbH, Erlangen, Germany) running the commercially available InSpace 3D software (Siemens Healthcare GmbH, Erlangen, Germany) and HS-FD-CT data was reconstructed within the Artis Icono platform (Siemens Healthcare GmbH, Erlangen, Germany). For both systems, the same reconstruction parameters were used (kernel type “HU,” image impression “sharp,” matrix 512 × 512, and isotropic voxel size 0.15 mm).

MS-CT data was postprocessed using the J70h bone kernel.

Next, triplanar multiplanar reformations (MPR) aligned to the cochlea, the CI, and the stapes were compiled with a slice thickness and distance of 0.15 mm each for the FD-CT datasets and of 0.5 mm each for the MS-CT datasets.

**Fig. 3:**
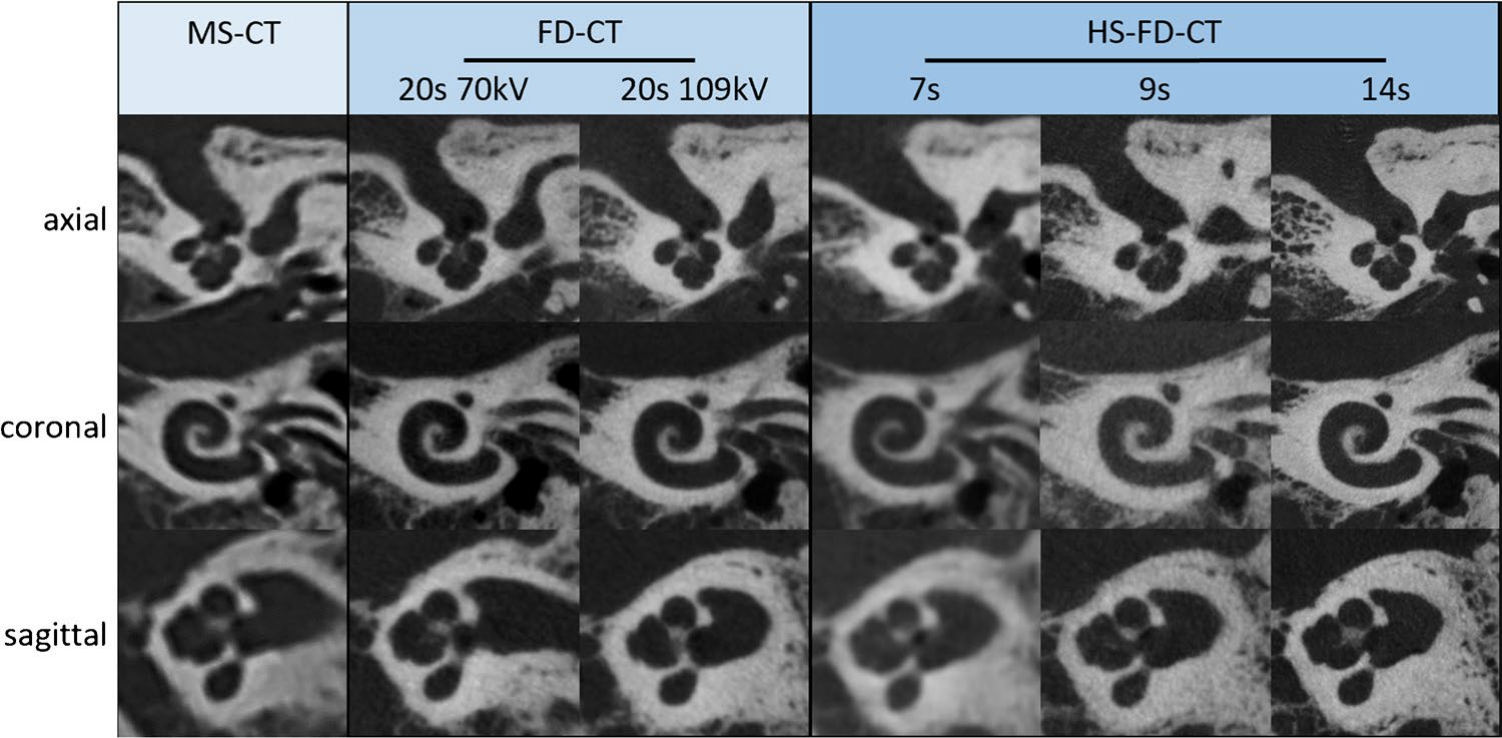
Exemplary MS-CT, FD-CT, and HS-FD-CT images aligned to the cochlea of the temporal bone specimen without a CI comparing different scanning protocols

**Fig. 4:**
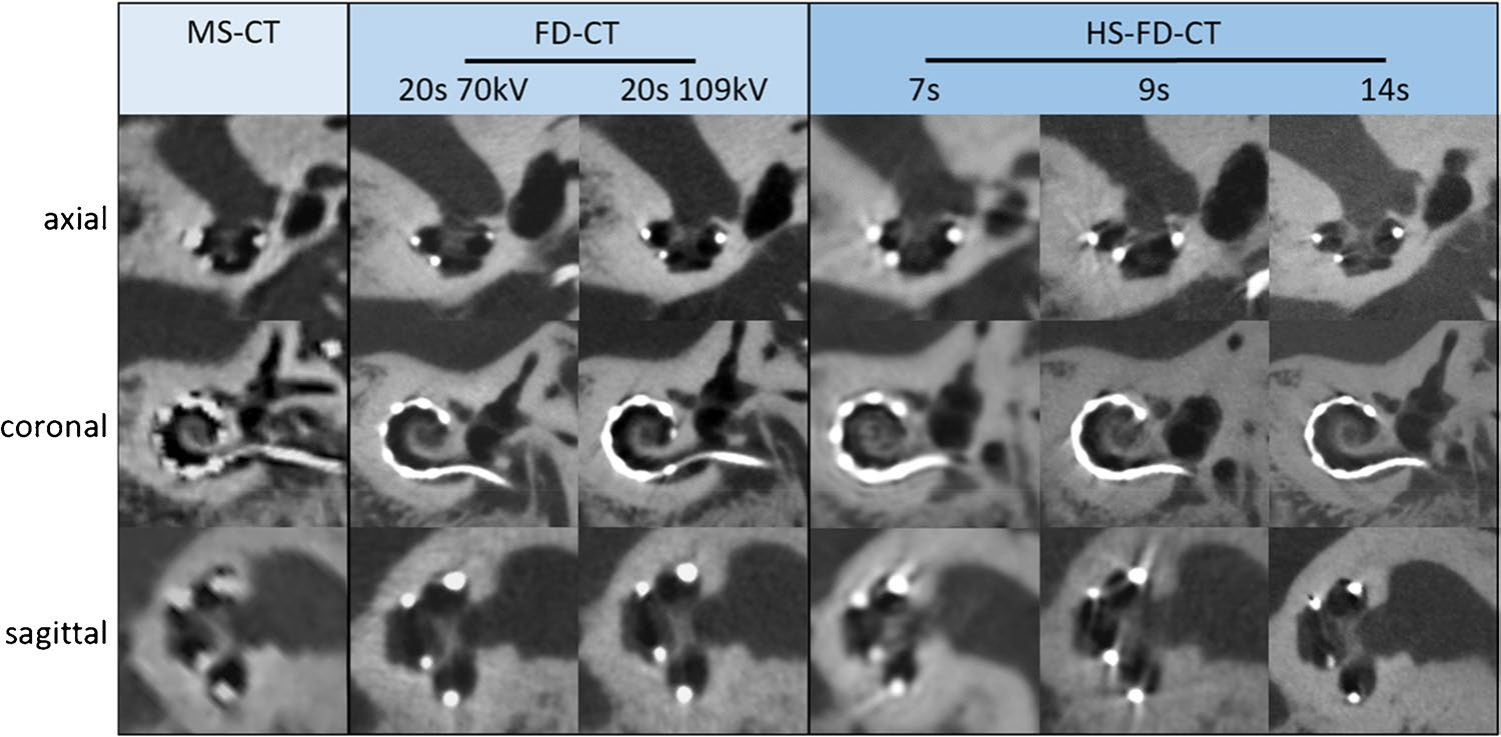
Exemplary MS-CT, FD-CT, and HS-FD-CT images aligned to the cochlear implant of the temporal bone specimen with a CI comparing different scanning protocols

**Fig. 5:**
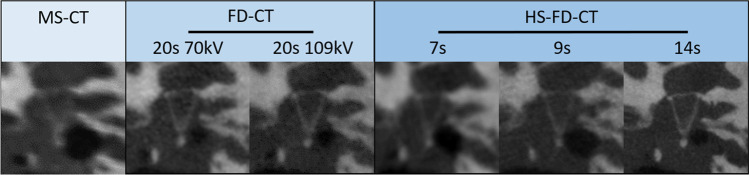
MS-CT, FD-CT, and HS-FD-CT MRPs aligned to the stapes comparing different scanning protocols

**Fig. 6:**
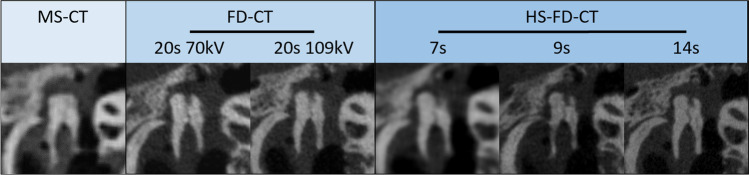
MS-CT, FD-CT, and HS-FD-CT MRPs aligned to the incudomalleolar joint comparing different scanning protocols

**Fig. 7:**
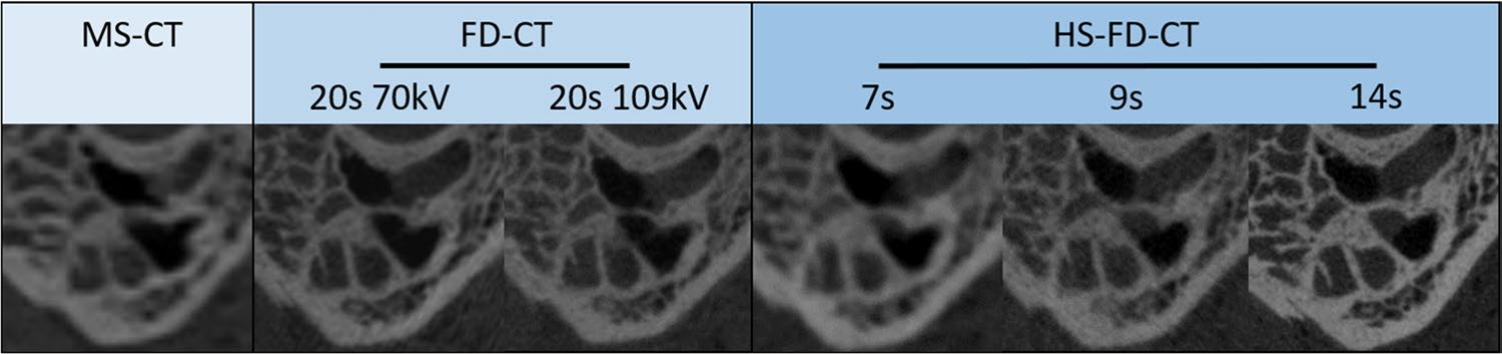
MS-CT, FD-CT, and HS-FD-CT MRPs of the mastoidal cells comparing different scanning protocols

Exemplary images for both MS-CT and the two different FD-CT systems and all scanning protocols are presented in Fig. 3 (specimen TB_0_), Fig. 4 (specimen TB_1_), Fig. 5 (MPRs aligned to the stapes), Fig. 6 (MPRs aligned to the incudomalleolar joint), and Fig. 7 (MPRs through the mastoid cells).

### Data evaluation

All datasets were analysed with the commercially available viewing software (syngo.plaza, Siemens Healthcare GmbH, Erlangen, Germany) in consensus reading by two experienced neuroradiologists.

#### Image quality

All datasets were evaluated for parameters that could compromise the IQ in consensus reading by using a fourfold scaled grading system derived from Struffert et al. [17] and modified to enable precise assessment of the IQ. The following parameters were rated: cochlea delineation, lamina spiralis ossea visibility, CI integrity, distinction of single CI electrodes, determination of intracochlear implant position, metal artifacts, homogeneity of the bony structures, malleus, incus, and stapes as well as Fallopian canal and semicircular canal delineation and the mastoid cell septation (see also Table 2). To determine the overall best IQ, the sum of all parameters was computed for each system and scanning protocol.

**Table 2:** Evaluated image quality parameters modified and derived from Struffert et al. [17]

	0	1	2	3
Cochlea delineation	Not distinguishable	Severely compromised by artifacts or blurring	Minimal artifacts, good visibility of cochlear details	No artifacts, excellent delineation of cochlear details
Lamina spiralis ossea visibility	Not visible	Partially visible	Visible in most parts of the cochlea	Good delineation of the lamina
CI integrity	CI not visible	CI visible, no tip fold	-	-
Distinction of single CI electrodes	Not distinguishable	Blurred, single electrodes can only be suspected	Single electrodes visible, severe artifacts	Single electrodes clearly visible, minimal artifacts
Determination of the CI position	Electrode within the cochlea, no other details can be seen	The position of the electrodes relative to the lamina spiralis ossea can partially be suspected	The position of the electrodes relative to the lamina spiralis ossea can be suspected in most cochlea parts	Electrode can be determined relative to the lamina spiralis ossea
Metal artifacts	No diagnostic value (e.g. because cochlea completely blurred)	Severe metal artifacts	Minimal metal artifacts, minimal blurring,	Without relevant metal artifacts
Homogeneity of the bony structures	Obvious noise, bony edges blurred	Severe noise, bony edges partially blurred	Minimal noise, minimal blurred bony edges	Noise barely seen, bony edges sharp
Malleus delineation	Malleus not visible	Strongly blurred	Malleus minimal blurred	Sharp delineation of the malleus
Incus delineation	Incus not visible	Incus strongly blurred	Incus minimal blurred	Sharp delineation of the incus
Stapes delineation	Stapes not visible	Stapes can partially be suspected	Stapes minimal blurred	Sharp delineation of the stapes
Fallopian canal delineation	Fallopian canal not visible	Fallopian canal partially strongly blurred	Fallopian canal minimal blurred	Sharp delineation of the Fallopian canal
Semicircular canal delineation	Semicircular canals not visible	Semicircular canals strongly blurred	Semicircular canals minimal blurred	Sharp delineation of all semicircular canals
Mastoid cell septation	No septation differentiable	Severe blurring of the mastoid cells	Minimal blurring of the mastoid cell septation	Sharp delineation of the mastoid cell septation

#### Radiation dose

To compare the dose characteristics of the different systems and the applied scanning protocols, the volume CT dose index (CTDI_vol_, mGy) was derived from the dose report provided by the scanners. The dose length product (DLP, mGycm) was computed as ${CTDI}_{vol}(\mathrm{mGy})\times \mathrm{scan length }\left(\mathrm{cm}\right)$ as described in similar studies [17, 23].

### Statistical analysis

Statistical analysis was performed with Excel (Microsoft, Redmond, USA).

## Results

### *Image quality (see also Table **3**)*

**Table 3:** Evaluation of the image quality parameters for MS-CT and the different (HS-)FD-CT protocols

	Cochlea delineation	Lamina spiralis ossea visibility	CI integrity	Distinction of single CI electrodes	Determination of the intracochlear implant position	Metal artifacts	Homogeneity of the bony structures
MS-CT	1	0	1	1	0	1	0
FD-CT 20 s 70 kV	2	1	1	3	2	3	2
FD-CT 20 s 109 kV	2	1	1	3	2	3	2
HS-FD-CT 7 s	1	0	1	2	1	1	1
HS-FD-CT 9 s	2	1	1	2	2	2	2
HS-FD-CT 14 s	3	2	1	3	2	3	3
	Malleus delineation	Incus delineation	Stapes delineation	Fallopian canal delineation	Semicircular canal delineation	Mastoid cell septation	In total
MS-CT	1	1	1	1	2	1	11
FD-CT 20 s 70 kV	2	2	2	2	3	2	27
FD-CT 20 s 109 kV	2	2	2	2	3	2	27
HS-FD-CT 7 s	1	1	1	1	2	1	14
HS-FD-CT 9 s	2	2	2	2	3	2	25
HS-FD-CT 14 s	3	3	3	3	3	3	35

*MS-CT*, multislice CT; *FD-CT*, flat-detector computed tomography; *HS-FD-CT*, high-speed flat-detector computed tomography.

IQ parameters were rated the highest with “35” in the 14 s HS-FD-CT datasets, followed by a rating of “27” in the 20 s 70 kV and 20 s 109 kV FD-CT datasets. 9 s HS-FD-CT datasets were rated with a total of “25”, 7 s HS-FD-CT datasets were rated with a total of “14”. IQ parameters were rated the lowest with “11” in the MS-CT.

IQ regarding cochlea delineation, homogeneity of the bony structures, malleus, incus, and stapes, as well as fallopian canal delineation and mastoidal cell septation, was rated the highest with “3” in the 14 s HS-FD-CT datasets. IQ regarding lamina spiralis ossea visibility was rated the highest with “2” in the 14 s HS-FD-CT datasets. IQ regarding distinction of single CI electrodes and metal artifacts was rated the highest with “3” in the 14 s HS-FD-CT, the 20 s 70 kV and the 20 s 109 kV datasets. IQ regarding delineation of semicircular canals was rated the highest with “3” in the 9 s and 14 s HS-FD-CT, the 20 s 70 kV and the 20 s 109 kV datasets. IQ regarding determination of the intracochlear implant position was rated the highest with “2” in the 14 s HS-FD-CT, the 20 s 70 kV and the 20 s 109 kV datasets.

IQ regarding determination of the intracochlear implant position and homogeneity of the bony structures was rated the lowest with “0” in the MS-CT. IQ regarding lamina spiralis ossea visibility was rated the lowest with “0” in the MS-CT and the 7 s HS-FD-CT datasets. IQ regarding distinction of single CI electrodes was rated the lowest with “1” in the MS-CT. IQ regarding cochlea delineation, metal artifacts, malleus, incus, stapes, and Fallopian canal delineation and mastoid cell septation was rated the lowest with “1” in the MS-CT and the 7 s HS-FD-CT datasets. IQ regarding delineation of semicircular canals was rated the lowest with “2” in the MS-CT and the 7 s HS-FD-CT datasets.

Table 3 summarizes IQ evaluation of MS-CT, FD-CT, and HS-FD-CT.

### Radiation dose (see also Tables 4 and 5)

**Table 4:** Radiation dose of MS-CT, FD-CT, and HS-FD-CT in the temporal bone specimen without a CI

	CTDI_vol_ (mGy)	DLP (mGycm)
MS-CT	27.5	138
FD-CT 20 s 70 kV	49.4	247
FD-CT 20 s 109 kV	38.3	192
HS-FD-CT 7 s	37.5	188
HS-FD-CT 9 s	40.7	204
HS-FD-CT 14 s	166	829

*MS-CT*, multislice CT; *FD-CT*, flat-detector computed tomography; *HS-FD-CT*, high-speed flat-detector computed tomography.

**Table 5:** Radiation dose of MS-CT, FD-CT, and HS-FD-CT systems in the temporal bone specimen with a CI

	CTDI_vol_ (mGy)	DLP (mGycm)
MS-CT	27.5	138
FD-CT 20 s 70 kV	78.1	391
FD-CT 20 s 109 kV	56.6	283
HS-FD-CT 7 s	60.4	302
HS-FD-CT 9 s	56.6	283
HS-FD-CT 14 s	191	956

*MS-CT*, multislice CT; *FD-CT*, flat-detector computed tomography; *HS-FD-CT*, high-speed flat-detector computed tomography.

MS-CT showed the lowest CTDI_vol_ for both temporal bone specimens (CTDI_vol MS-CT TB0_ = 27.5 mGy; CTDI_vol MS-CT TB1_ = 27.5 mGy). The 14-s scanning protocol of the HS-FD-CT showed the highest CTDI_vol_ for both temporal bone specimens (CTDI_vol 14 s TB0_ = 166 mGy; CTDI_vol 14 s TB1_ = 191 mGy).

MS-CT showed the lowest DLP for both temporal bone specimens (DLP_MS-CT TB0_ = 138 mGycm; DLP_MS-CT TB1_ = 138 mGycm). The 14-s scanning protocol of the HS-FD-CT showed the highest DLP for both temporal bone specimens (DLP_14s TB0_ = 829; DLP_14s TB1_ = 956).

Tables 4 and 5 summarize the radiation exposure of MS-CT, FD-CT, and HS-FD-CT for both temporal bone specimens.

## Discussion

Here we compared MS-CT and two different generations of angiography-mounted FD-CT systems (standard FD-CT and high-speed FD-CT) using different scanning protocols regarding IQ (delineation of the bony structures of the inner ear and single CI electrodes, the evaluation of the intracochlear implant position, the susceptibility for metal artifacts), the associated radiation exposure, and scan time.

In agreement with several previous studies [13, 16–18] and due to their superior spatial resolution and low susceptibility for metal artifacts, both FD-CT systems yield better image quality than MS-CT for evaluation of the microanatomical details of the temporal bone and the precise cochlear implant position: in our study, the HS-FD-CT with its 14-s scanning protocol provided the best visualization of the temporal bone specimens and the CI, followed closely by the FD-CT using the 20 s 70 kV and 109 kV protocol and the 9 s HS-FD-CT protocol. Although providing worst IQ among tested (HS-)FD-CT protocols, the 7 s HS-FD-CT protocol still exceeds MS-CT. In contrast, both FD-CT systems had higher CTDI_vol_ and DLP compared to MS-CT—regardless of the applied scanning protocol. This is in accordance to previous studies reporting effective doses for angiography-mounted FD-CT systems up to twice as high as for MS-CT [19, 23, 24]. In our study, the 14-s scanning protocol of the HS-FD-CT presented with the highest CTDI_vol_ and DLP among the tested scanners and protocols. The other tested FD-CT scanning protocols presented with DLPs in a comparable range of 188 to 247 mGycm for the specimen without a CI and 283 to 391 mGycm for the specimen with a CI. Regarding scan time, MS-CT is still the modality with the shortest scan time (5 s), yet the HS-FD-CT system significantly reduces the scan time up to 65% compared to FD-CT (20 s in FD-CT versus 7 s in HS-FD-CT); even the longest HS-FD-CT protocol (14 s) still shortens scan time by 6 s compared to both FD-CT protocols. This extensive scan time reduction is possible due to the higher framerate of the HS-FD-CT detector and the consecutive faster C-arm rotation. Especially uncooperative, moving patients should benefit from this significantly reduced scan time of HS-FD-CT.

Whereas each tested (HS-)FD-CT protocol provided diagnostic images, there were relevant differences regarding image quality, diagnostic information, and scan time. Thus, it is the radiologist’s responsibility to tailor the characteristics of the (HS-)FD-CT protocols to the clinical requirements. Therefore—based on our experience—we want to recommend scanning protocols for three different clinical settings:

- 1.) postoperative CI control in a cooperative patient: for this common situation, we recommend the 9 s HS-FD-CT scanning protocol combining excellent IQ, short scan time, and acceptable radiation exposure allowing reliable assessment of the CI and its intracochlear position. If available, dedicated high-end CBCT should be an excellent alternative in these patients, and this with much lower CTDI_vol_/DLP (less than half of the radiation exposure used on MS-CT).
- 2.) microanatomical temporal bone assessment in a cooperative patient (e.g. for detection or exclusion of otosclerotic lesions or a CI insertion trauma): in this challenging scenario, we recommend the 14 s HS-FD-CT scanning protocol providing the highest diagnostic value of all tested protocols, yet as this protocol is associated with the highest radiation exposure, its application should be responsibly indicated. If available, CBCT should be an excellent alternative in these patients at a much lower radiation exposure.
- 3.) postoperative CI control in an uncooperative patient with expected motion artifacts: in this worst-case scenario, we recommend the 7 s HS-FD-CT scanning protocol providing sufficient diagnostic information combined with short acquisition time. MS-CT might be an adequate imaging alternative in these patients due to its short scan time but with significantly lower image quality. Both techniques are an alternative in uncooperative patients for the slower dedicated CBCT systems.

Our study has some limitations: first, CTDI_vol_ and DLP values were derived from the scan reports provided by the MS-CT and FD-CT systems; no phantom was used to measure the effective dose. However, the CTDI_vol_ and DLP values are suggestive of the different radiation exposures. Nonetheless, actual phantom measurements are needed to verify our findings regarding radiation dose. Second, our results are based on two scanned specimens without actual testing in patients. Thus, image quality of (HS-)FD-CT and MS-CT may vary in vivo, especially due to possible motion artifacts, and further studies are needed to assess the diagnostic value of HS-FD-CT in the clinical routine. Third, no comparison of the angiography-mounted (HS-)FD-CT and a dedicated CBCT system regarding image quality and scan time was performed. Because of its affordable price compared to angiography systems, lighter and smaller equipment, better spatial resolution compared to MS-CT and equal spatial resolution compared to FD-CT, the simplicity to operate and maintain, the possibility to scan seated patients and the open design to help claustrophobic patients [25], CB-CT is the preferred imaging modality for dental and maxillofacial skeleton imaging [25] and is also applicable for temporal bone imaging as well as postoperative cochlear implant control. In this context, CB-CT significantly reduces radiation dose compared to MS-CT and FD-CT [26]: for example, in a recent study, Helal et al. report a median DLP of 93 mGycm for CBCT in comparison to a median DLP of 387.5 mGycm for MS-CT [13]. In this context, Burck et al. measured the effective dose of CBCT in comparison to MS-CT and found an effective dose reduction of up to 97.7% via CBCT [12]. However, to the best of our knowledge, there are no studies comparing image quality of CBCT and (HS-)FD-CT for temporal bone imaging. Fourth, our results are exclusively based on a singular specific CI model and performance of (HS-)FD-CT and MS-CT may be more challenging in patients with other cochlear implants featuring smaller electrode contacts or smaller intercontact distances. Fifth, the acquisition of (HS-)FD-CT datasets with a slice thickness of 0.06 mm can result in a high level of noise. To decrease the noise and obtain excellent image quality, higher doses are then required. Further studies are needed to determine the optimal balance of spatial resolution, scan dose, and signal-to-noise ratio in HS-FD-CT.

## Conclusion

HS-FD-CT improves visualization of the temporal bone anatomy and the postoperative CI assessment in comparison to standard, slow-rotating angiography-mounted FD-CT systems by combining higher or equal image quality, faster scan time, and a comparable radiation exposure. However, it is the radiologist’s responsibility to tailor the characteristics of the (HS-)FD-CT protocols to clinical requirements. Despite the higher radiation exposure in comparison with MS-CT and the slower, dedicated CBCT systems, the major potential of HS-FD-CT is the reduction of artifacts in uncooperative patients.

## Data Availability

Not applicable.
